# The novel compound PBT434 prevents iron mediated neurodegeneration and alpha-synuclein toxicity in multiple models of Parkinson’s disease

**DOI:** 10.1186/s40478-017-0456-2

**Published:** 2017-06-28

**Authors:** David I. Finkelstein, Jessica L. Billings, Paul A. Adlard, Scott Ayton, Amelia Sedjahtera, Colin L. Masters, Simon Wilkins, David M. Shackleford, Susan A. Charman, Wojciech Bal, Izabela A Zawisza, Ewa Kurowska, Andrew L. Gundlach, Sheri Ma, Ashley I. Bush, Dominic J. Hare, Philip A. Doble, Simon Crawford, Elisabeth CL. Gautier, Jack Parsons, Penny Huggins, Kevin J. Barnham, Robert A. Cherny

**Affiliations:** 1 The Florey Institute of Neuroscience and Mental Health, The University of Melbourne, Melbourne, VIC 3010 Australia; 2 Prana Biotechnology Ltd, Parkville, VIC 3052 Australia; 3 Centre for Drug Candidate Optimisation, Monash Institute of Pharmaceutical Sciences, Monash University, Parkville, VIC 3052 Australia; 4 The Institute of Biochemistry and Biophysics, Polish Academy of Sciences, Warsaw, Poland; 5 Elemental Bio-imaging Facility, The University of Technology Sydney, Broadway, Ultimo, NSW 2007 Australia; 6 Australia Electron Microscope Unit, School of Biosciences, The University of Melbourne, Melbourne, VIC 3010 Australia; 7 Bio21 Institute and Department of Pharmacology and Therapeutics, The University of Melbourne, Melbourne, VIC 3010 Australia

**Keywords:** Synucleinopathy, Drug development, Chelation, Oxidative stress, Neuroprotection

## Abstract

Elevated iron in the SNpc may play a key role in Parkinson’s disease (PD) neurodegeneration since drug candidates with high iron affinity rescue PD animal models, and one candidate, deferirpone, has shown efficacy recently in a phase two clinical trial. However, strong iron chelators may perturb essential iron metabolism, and it is not yet known whether the damage associated with iron is mediated by a tightly bound (eg ferritin) or lower-affinity, labile, iron pool. Here we report the preclinical characterization of PBT434, a novel quinazolinone compound bearing a moderate affinity metal-binding motif, which is in development for Parkinsonian conditions. In vitro, PBT434 was far less potent than deferiprone or deferoxamine at lowering cellular iron levels, yet was found to inhibit iron-mediated redox activity and iron-mediated aggregation of α-synuclein, a protein that aggregates in the neuropathology*.* In vivo, PBT434 did not deplete tissue iron stores in normal rodents, yet prevented loss of substantia nigra pars compacta neurons (SNpc), lowered nigral α-synuclein accumulation, and rescued motor performance in mice exposed to the Parkinsonian toxins 6-OHDA and MPTP, and in a transgenic animal model (hA53T α-synuclein) of PD. These improvements were associated with reduced markers of oxidative damage, and increased levels of ferroportin (an iron exporter) and DJ-1. We conclude that compounds designed to target a pool of pathological iron that is not held in high-affinity complexes in the tissue can maintain the survival of SNpc neurons and could be disease-modifying in PD.

**Electronic supplementary material:**

The online version of this article (doi:10.1186/s40478-017-0456-2) contains supplementary material, which is available to authorized users.

## Introduction

Idiopathic Parkinson’s disease (PD) is characterized by loss of the neurons of the *substantia nigra pars compacta* (SNpc) and the presence of intracellular Lewy bodies composed primarily of fibrillar deposits of the synaptic protein α-synuclein. The physiological function of α-synuclein is poorly understood, but genetic evidence strongly implicates this highly-expressed protein in the pathological process in PD and other movement disorders such as Multiple System Atrophy and Diffuse Lewy Body Disease, (classified as *synucleinopathies*).

α-synuclein may be neurotoxic in the fibrillar state or soluble oligomeric species [20, 42]. However, it is expressed throughout the brain, so the selective vulnerability of the SNpc in PD indicates other local factors may be involved in toxicity. This could be due to the co-localization within the SNpc of the chemical reductant dopamine (DA) with an abundance of the redox active metal iron, in addition to α-synuclein [41, 43]. α-synuclein binds iron with micromolar affinity [22], and both iron and DA promote the aggregation of α-synuclein and the formation of Lewy bodies [15, 16, 28, 33], and can react together to form damaging peroxides [40]. There is consistent evidence of a regional increase of iron in the SNpc of PD patients [10, 25, 64] as well as a rapid accumulation of nigral iron in animals following intoxication with the Parkinsonian agents 6-hydroxydopamine (6-OHDA) and 1-methyl-4-phenyl-1,2,3,6-tetrahydropyridine (MPTP) [27, 70]. Iron is needed for numerous catalytic and metabolic processes including as a cofactor for tyrosine hydroxylase (TH), the enzyme responsible for the production of L-3,4-dihydroxyphenylalanine (L-DOPA) from tyrosine [36, 85]. However, dysregulation of iron through aging may increase the potential for oxidative damage [11], which is exaggerated in the SNpc in PD [9].

Recently, the high affinity iron chelator deferiprone (3-Hydroxy-1,2-dimethyl-4-pyridinone) was reported to provide benefit in a phase 2 randomized clinical trial in PD patients [24]. Although deferiprone was well tolerated there remain concerns with known adverse effects of strong chelators [81]. Indeed, there is no certainty about the compartment of pathological iron in the SNpc that might mediate the neuropathology. Since the labile iron pool is not tightly bound, we reasoned that it might be possible to provide neuro-rescue with moderate-affinity bioavailable iron chelators. We have been developing candidate drugs for neurodegenerative diseases designed to re-establish normal metal homeostasis and abort the oligomerization of susceptible proteins caused by pathological low affinity adventitial metal ligation [1, 2, 18, 53, 57]. PBT434, an orally bioavailable 8-hydroxyquinazolin-4(3H)-one, binds iron sufficiently to abolish pathological reaction with α-synuclein, but with an affinity that is designed not to disrupt physiological iron homeostasis.

We hypothesized that targeting the pathological pool of nigral iron that emerges in these models with PBT434 would preserve function and the consequent accumulation of α-synuclein. We tested PBT434 for its ability to preserve neuronal viability and connectivity, motor function, α-synuclein accumulation and markers of oxidative stress in three animal models of PD: the Parkinsonian toxins 6-OHDA and MPTP, and hA53T transgenic mice (which overexpress human α-synuclein bearing the alanine to threonine mutation at position 53, which causes familial PD).

## Materials and methods

### Potentiometry

Potentiometric titrations of the peptides were performed on a MettlerTitrando 907/Dosino 800 titration system, using InLab 422 combined glass-Ag/AgCl electrodes (Mettler-Toledo), which were calibrated daily by nitric acid titrations [2]. 0.1 M NaOH (carbon dioxide free) was used as titrant. Sample volumes of 1.2–1.5 ml were used. The samples contained typically 0.8 mM PBT-434, dissolved in 4 mM HNO3/96 mM KNO3. The Fe (II) and Fe (III) complex formation was studied using a 2.5–4-fold excess of the compound over the metal ion, added as nitrate. All experiments were performed under argon at 25 °C, in the pH range of 2.3 to 12.2. The collected data were analyzed using the HYPERQUAD program [1]. Three to five titrations were included simultaneously into calculations, separately for protonation, Fe (II) and Fe (III) complexation.

The UV-visible spectra were recorded at 25 °C on a Cary 50 or a Perkin Elmer spectrophotometer, over the spectral range of 230–800 nm. The optical path for all experiments was 1 cm. The samples containing PBT-434 alone or with Fe (II), Fe (III), Cu (II) or Zn (II) ions were titrated with NaOH in the pH range of 2.0–12.0, by careful manual additions of very small amounts of the concentrated base solution. For Fe (III) and Fe (II) the PBT-434 concentration used was 0.1 mM, and the ligand-to-metal ratio was 4:1, to keep in line with conditions that delivered good potentiometic titrations. For Cu (II) the PBT434 concentration used was 0.1 mM, and the ligand-to-metal ratios used varied between 1:1 and 4:1. For Zn (II) spectroscopic titrations were performed at a lower concentration of 0.04 mM PBT434 and 0.02 mM Zn (II) to avoid precipitation. The Fe (II) samples were prepared under nitrogen, in a Coy glove box, and transferred to the spectrophotometer [34, 46].

### Inhibition of metal/dopamine mediated H_2_O_2_ generation

This technique, adapted from established protocols [74], is a dicholorofluoroscein (DCF)-based fluorometric assay that evaluates the ability of a test compound to inhibit H_2_O_2_ generated by redox active metals in the presence of a reducing agent.

### α-synuclein aggregation assay

Each batch of recombinant α synuclein that was synthesised underwent protein sequencing and mass spectrometry to ensure purity at the Monash Protein Production Unit (Monash University, Australia). The lyophilised purified WT recombinant α synuclein was reconstituted with Tris Buffer Saline (TBS) pH 7.4. Pooled aliquots were spun at 100,000 g for 30 mins at 4° to remove pre-formed aggregates/seeds. The supernant containing the monomeric form was collected and used in the assay. The protein concentration was determined using BCA method Iron Nitrate was weighed and dissolved in TBS solution. PBT434 was dissolved in 100% DMSO, then diluted to stock solution using milliQ water. To each tube, TBS, Fe, Compound/Veh then α synuclein was added in sequence with equal concentrations. The final concentration of α synuclein, Fe and compound was 186.6 μM**.**

Once all solutions were in the tubes, samples were vortex for 2 s before plating up. Samples were assayed in the presence of ThT (20 μM). The assay was read in a Perkin-Elmer Enspire multi-mode plate reader set at 37°, reading every 30 mins (1800 s), shaking at 800 rpm (1800 Seconds) between each read up to 42 h. ThT fluorescence intensity was measured over time at wavelengths 450 emission and 485 nm excitation. The RFU values were normalised to TBS ThT blank wells and were plotted over time. The lag-time and the maximal relative fluorescent units (RFU) were reported as a measure of kinetic profiling of compounds. These were calculated based on a 4-point parameter sigmoidal curve (plotted in Sigmaplot V12.5).

### Preparation of α-synuclein fibril samples for transmission electron microscopy

Forty-two hours after initiating the α-synuclein reaction 20uL droplets were adsorbed onto formvar-coated copper grids for 30 mins. After incubating the excess solution was blotted away and the samples on grids were stained with 1% uranyl acetate for 30 s. The excess stain was then blotted away and the grids allowed to dry at room temperature overnight. The samples on grids were viewed in an FEI Tecnai Spirit transmission electron microscope at 120 kV. Images were captured with a Gatan Eagle digital camera at a resolution of 2 K × 2 K pixels.

### Iron efflux assay

M17 neuroblastoma cells were detached from two 175 cm flasks and re-suspended in 100 ml of Optimem growth medium. Cells were transferred to poly-d-lysine coated, 12-well plates and allowed to recuperate for 24 h. 50 μl of ^59^Fe was added to 800 μl of Optimem (without serum) for overnight incubation. Cells were then washed in HBSS (buffered saline) × 3, then 500 μl of HBSS was added with either no drug 1 μM, 10 μM, 20 μM of PBT434 or deferiprone. Experiment was terminated after 3 h. Medium was removed and radiation measured using a gamma counter.

### Mice

Male C57BL/6 J mice aged 12 weeks and weighing ≈25 g were used for the 6-OHDA and MPTP studies (Animal Resources Centre; Western Australia). The hA53T Tg mice were bred in-house (Jax Stock No: 004479; B6;C3-Tg (Prnp-SNCA*A53T)83Vle/J; A53T α-synuclein transgenic line M83). PBT434 (at 30 mg/kg/day) was delivered either by oral gavage or by being mixed into rodent chow (Glen Forrest Stockfeeds, Western Australia; spiked at 0.25 g/kg food; 20 days days of treatment for the MPTP; 18 days of treatment 6-OHDA; four months of daily treatment for the hA53T Tg mice).

### 6-OHDA intoxication model

Mice anesthetized with 2.5–3% isoflurane were placed into a stereotaxic apparatus and 3.0 μg of 6-OHDA was injected into the right SNpc, as described in [77]. Amphetamine induced (5 mg/kg) rotational behavior was measured three days after 6-OHDA lesion using an automated Rotacounter system (Columbus Instruments, Columbus, OH, USA). Robust rotational behavior has been observed as early as one-day post-lesion [82, 87]. Only mice that exhibited rotations at day 3 between 200 and 450 times per hour were included in the trial. Mice were then randomly assigned to the PBT434 treatment group or sham-vehicle (VEH) treatment group. The PBT434 treatment group was gavaged at 30 mg/kg/day, commencing 3 days following induction of lesion. Experimenters were blinded to the assignment of treatments for each of the groups. Mice were retested and then culled twenty-one days post 6-OHDA lesion.

### MPTP model

Mice were administered an acute dosing regimen of four injections of MPTP (Sigma, USA) two hours apart [4, 5, 61]. Each experimental trial contained MPTP lesioned animals that were randomly subdivided into a sham treated group (vehicle alone) and drug treatment group (30 mg/kg/day PBT434, commencing 24 h after MPTP until culled at day 21). Experimenters were blinded to the assignment of treatments for each of the groups. In one group of animals the mice were treated with analog of PBT434 (PBT434-met 30 mg/kg/day) which does not have the ability to bind metals as a control.

The pole test was used to measure motor co-ordination and performance at twenty days post MPTP injection [32, 67, 73]. The mice were assessed on their ability to rotate their heads and their bodies 180° to position themselves in order to turn down descend down the pole to the home cage [44]. The fastest times obtained from the five trials were used as the value for turn time; measured in seconds. Experimenters were blinded to the treatments of each of the groups.

### Hindlimb clasping and motor activity

Briefly, mice were suspended by the base of the tail and their behaviors were recorded for 30 s [62, 78]. Spontaneous motor activity was measured in the hA53T α- synuclein mouse using an open field photo-beam activity System (Truscan 2.0, Coulbourn Instruments, USA) [61].

### Tissue preparation

At the completion of a trial, mice were killed with an overdose of anesthetic, perfused with phosphate buffered saline (PBS) pH 7.4 and tissues collected [61]. The blood samples were analyzed by a veterinary pathology service (Gribbles Veterinary Australia, accreditation to ISO/IEC 17025 conferred by the National Association of Testing Authorities and certification to AS/NZS 9001: 2008 conferred by Lloyd’s Register Quality Assurance).

### Western blots

Western blot was used to investigate protein expression of human α- synuclein in the hA53T mouse (LB509; 1:10,000; Abcam, USA), the expression of endogenous α- synuclein (97/8 1;10,000; a gift from Dr. Janetta Culvenor, University of Melbourne, Australia) or human, tyrosine hydroxylase (TH, 1:10,000; Millipore, USA), Protein deglycase, also known as Parkinson disease protein 7 (DJ-1, Polyclonal, 2134, CST, Boston, USA), Ferroportin-1 (1:1000; Santa Cruz, USA), Anti-synaptophysin, clone SY38 (1:10,000; Millipore, USA), Samples were homogenised in PBS buffer (PBS, Invitrogen, EDTA-free Protease cocktail inhibitor, Roche, USA) with 11.02 mg/mL BHT (Sigma-Aldrich, USA) in acetonitrile (Sigma-Aldrich, USA) solution using sonication (20% amplitude, pulses 1–2 s while on ice). The crude homogenate was spun on a benchtop centrifuge for 10 mins 4 °C. Homogenate was assayed for protein (Thermo Scientific Pierce BCA Protein Assay Kit) and between 5 and 10 μg of protein loaded onto NuPage Novex bis-tris 4–12% 26 well gels (Invitrogen, USA). Total protein was measured on the membrane, with a ponceau stain (5 min at room temperature (0.1% ready se stain, Sigma-Aldrich, catalogue # P7170). Excess stain was washed from the membrane before exposing. The ponceau stain was used to normalise the protein of interest against total protein (loading control). The membrane was imaged using a LAS-3000 [1, 61].

### α -synuclein solubility analysis

Western blot for α- synuclein. Tissue samples were collected, frozen, and stored at −80 °C for later use. Brain homogenates were prepared by sequential extraction, with the 5% SDS soluble and 8 M urea soluble fractions examined by Western blot. Protein concentrations of the initial brain homogenates were estimated using an assay for bicinchoninic acid (BCA, Pierce Protein Assay Kit, ThermoFisher Scientific, U.S.A.). Western blots were probed with the α- synuclein antibody LB509 (Abcam, USA).

### 8-isoprostane ELISA

Dissected SN samples were collected, snap frozen and stored at -80°C until analysis. The tissue homogenates were analysed in the competitive ELISA as per the manufactures instructions (Cayman Chemicals).

### Metal analysis

Iron imaging of selected region (Substantia nigra; both pars compacta and pars reticulata) was performed on the 30 μm cryostat sections using Laser ablation-inductively coupled plasma-mass spectrometry (LA-ICPMS) on a New Wave Research UP213 laser ablation unit (Kennelec Scientific, Australia) coupled to an Agilent 7500ce ICP-MS system (Mulgrave, Australia). The full methods have been described previously [38, 59]. Additional iron measurements were performed using liquid phase ICPMS Varian UltraMass 700 (Varian, Australia) [12].

### Stereology

The total number of DA neurons in the SNpc was estimated using a fractionator sampling design [29, 61, 77]. Brains were fixed overnight in 4% paraformaldehyde in PBS, then cryoprotected in 30% sucrose until the brains sunk, the SNpc was sectioned in a 1 in 3 series at 30 μm with a cryostat (Leica), stained with the TH antibody, reacted with diamiobenzidine tetrahydrochloride (DAB; Sigma) and counter stained with neutral red [17]. Counts were made at regular predetermined intervals (x = 140 μm, y = 140 μm). Systematic samples of the area occupied by the nuclei were made from a random starting point. An unbiased counting frame of known area (45 μm × 35 μm) was superimposed on the image of the tissue sections using stereology software (MBF, Stereo Investigator) utilizing a 63 × objective lens (Leica, N.A.1.36). Experimenters were blinded to the treatments of each of the groups. Every neuron within the SNpc (TH – positive and negative) was counted to obtain an estimate of neuronal number. The density of TH-immunopositive varicosities was obtained from the dorsal 400 μm of the caudate putamen (CPu) [30].

### Cerebrospinal fluid collection from dogs

The collection of cerebrospinal fluid (CSF) was performed at the conclusion of a 28 day toxicology study in 10 month old Beagle dogs undertaken at Charles River Laboratories Preclinical services Edinburgh UK under appropriate ethics approval. PBT434 was administered by oral gavage once a day for 28 days at the following doses: vehicle control (0 mg/kg/day), 10 mg/kg/day, 30 mg/kg/day and 50 mg/kg/day. Each treatment arm included 3 male and 3 female dogs. The CSF was extracted at necropsy into collection tubes containing 10uL of butylated hydroxytoluene, frozen on dry ice and stored at −80 °C until analysis. Any samples showing signs of hemolysis were excluded due to the potential for contamination of α-synuclein from blood [52].

### Cerebrospinal fluid collection from rats

Cannulas were inserted into the lateral cerebral ventricles of wild-type rats by stereotactic surgery. CSF sampling was performed using a rodent microdialysis bowl (BASi, *n* = 8). Baseline CSF was sampled after which the animals were gavaged with PBT434 at 30 mg/kg. CSF samples were collected at one and four hours after gavage with PBT434. Samples were analysed by Western blot for the presence of α-synuclein as described.

### Statistical analysis

SPSS version 17.0 (Windows version), was used for all statistical analyses. Any significant differences in the mean scores were denoted by asterisks throughout the manuscript (* *p* < 0.05, ** *p* < 0.01 and *** *p* < 0.001).

## Results

### Affinity of PBT434 for metal ions

**Fig. 1:**
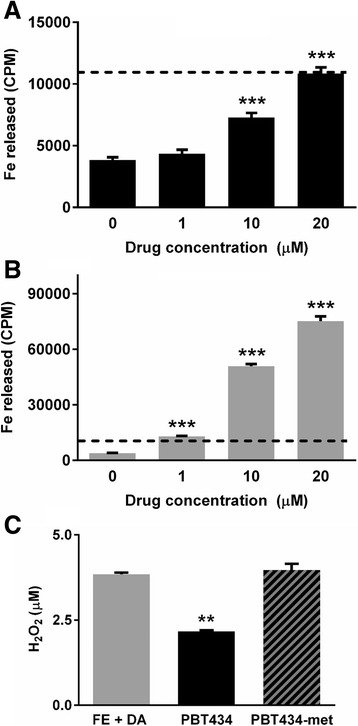
PBT434 enhances the release of iron and prevents the generation of hydrogen peroxide. Cultured M17 neuroblastoma cells were loaded with the iron isotope ^59^Fe. The cells were washed and then exposed to a chelator to assess if iron could be removed from the cell. Cells loaded with the iron isotope ^59^Fe were exposed to **a** PBT434 and the amount of radioactive ^59^Fe released into the media was measured (CPM = counts per minute) or **b** deferiprone at 0, 1, 10 or 20 μM for 3 h. Deferiprone showed a dose related increase in the levels of ^59^Fe secreted into growth medium. With PBT434 the effect was observed only at the highest dose of 20 μM (*P < 0.05, ** *P* < 0.01, *** *P* < 0.0001, One-way ANOVA, Tukey Post Hoc). At the highest concentration, the effect of deferiprone was 5-fold greater than for PBT434. The dashed line represents equivalent values on the two graphs. **c** PBT434 causes an inhibition of metal mediated redox activity. Fe-citrate (0.4 μM) in the presence of dopamine (DA, 50 mM) generate hydrogen peroxide (H_2_O_2_) assessed using a cell-free fluorescence-based assay. PBT434 at 10 μM but not PBT434-met significantly reduced H_2_O_2_ generated by Fe/DA (PBT434-met = analog of PBT434 in which the metal binding site is blocked; One-way ANOVA, Tukey Post Hoc). Dopamine without Fe-Citrate did not produce H_2_O_2_

PBT434 was found to have a dissociation constant (Kd) for Fe (III) and Cu (II) of ~10^−10^ M. The affinities for Fe (II) and Zn were found to be ~10^−5^ M and ~10^−7^ M respectively (Additional file 1: Data S1)*.* Classical high affinity iron chelators such as deferoxamine (Kd ≈ 10^−31^ M) and deferiprone (Kd ≈ 10^−35^ M) have been evaluated for their potential therapeutic use in iron overload conditions such as thalassemia and in PD [24, 75]. As PBT434 has greater than 20 orders of magnitude less affinity for iron than these classical chelators, it was thought valuable to evaluate whether the large difference in affinities might be reflected in the ability of the respective compounds to promote the efflux of Fe from biological tissues. Cultured neuronal M17 cells were exposed to a trace quantity of the gamma emitting iron isotope ^59^Fe and then to each compound over a range of concentrations. At the highest concentration (20 μM) PBT434 was shown to have a significant but ~5 fold lesser ability to promote the flow of Fe out of cultured neuronal M17 cells than 20 μM deferiprone (Fig. 1). When administered to normal unlesioned mice, PBT434 at the dose of 30 mg/kg/day for 21 days had no significant effect on brain iron levels or peripheral indices of iron trafficking and metabolism (Additional file 1: Figure S2).

### Inhibition of metal mediated redox activity

PBT434 was assessed for its ability to inhibit redox activity in an in vitro assay modeling an elevated dissociable pool of metals in the presence of the potent reductant dopamine (DA). Fe in the form of Fe (II)-citrate was incubated with DA in aerated buffer, and H_2_O_2_ production was assessed. PBT434 significantly inhibited H_2_O_2_ production by iron (Fig. 1c). An analog of PBT434 (PBT434-met) was synthesized in which the hydrogen of the phenolic hydroxide substituent was replaced by a methyl group, abolishing its ability to bind metals. The PBT434-met analog was unable to suppress H_2_O_2_ production (Fig. 1c).

### Iron mediated aggregation of α-synuclein

**Fig. 2:**
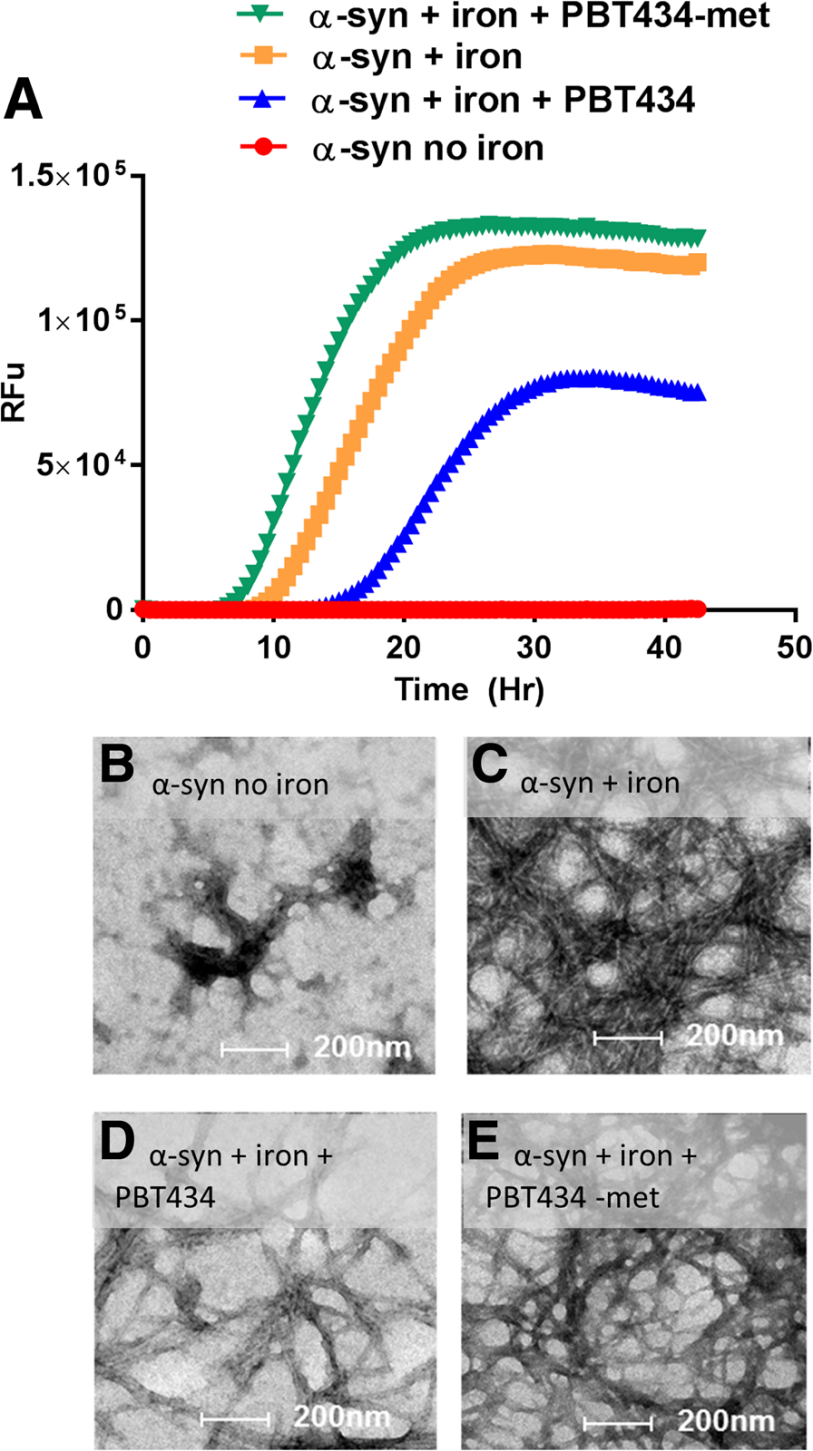
Inhibition of iron mediated α-synuclein (α-syn) aggregation. Recombinant α-synuclein (186.6 μM) was incubated alone or in the presence of equimolar concentrations of iron nitrate (iron = Fe (NO_3_)_3_), PBT434 or PBT434-met. Thioflavin T (ThT) fluorescence was measured (RFU = relative fluorescent units) every 30 min for 42 h. The lag-time of aggregation was slowed by PBT434 (α-synuclein = 37 h, α-syn + iron = 10.2 h, α-syn + iron + PBT434 = 16.60 h and α-syn + iron + PBT434-met =7.9 h). **a** One Way ANOVA with games-howell post hoc analysis (unequal variances on the lag-phases, individual reaction wells (*n* = 5–6) showed that, α-syn + iron had significantly faster aggregation than α-syn + iron + PBT434 (*p* = 0.02) or α-syn alone (*p* = 0.04) but not α-syn + iron + PBT434-met (*p* = 0.49). Lower panels: Electron micrographs of samples from each reaction mixture were taken after 42 h (**b**; α- synuclein, no iron); **c** Increased fibril formation in the presence of iron; **d** Diminished fibril formation in the presence of PBT434; E) PBT434-met did not reduce the fibril formation

In an in vitro assay modeling iron-mediated acceleration of α- synuclein aggregation, PBT434 significantly reduced the rate of Fe-mediated aggregation of α-synuclein as measured by the lag-time for the detection of fluorescent aggregates compared with α-synuclein/Fe alone. In contrast, PBT434-met did not inhibit the rate of Fe-mediated aggregation (Fig. 2), consistent with the aggregation being caused by Fe coordination.

### Neuroprotective effects of PBT434

**Fig. 3:**
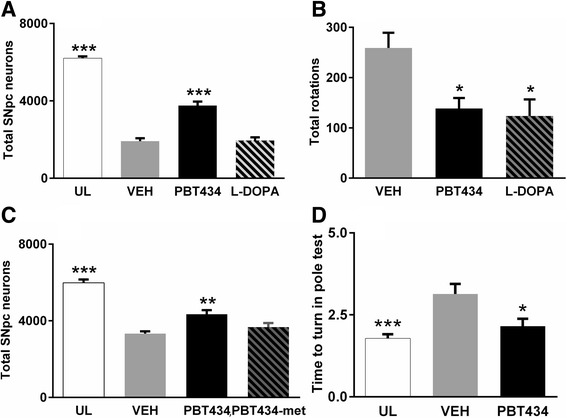
PBT434 prevents toxin induced cell loss and improves motor performance. **a** 6-OHDA injection resulted in the loss of 65% of the SNpc neurons compared with unlesioned control animals. PBT434 administration commencing 3 days following intoxication and significantly preserved neuron numbers compared with vehicle (*p* < 0.001, One-way ANOVA, Tukey Post Hoc). The number of neurons in an unlesioned mouse is 6124 ± 23. L-DOPA did not protect neurons against 6-OHDA toxicity. **b** Mice treated with PBT434 (30 mg/kg/day, *N* = 11 (*P* < 0.05) or L-DOPA (15 mg/kg, *P* < 0.001, One-way ANOVA, Tukey Post Hoc) showed significantly fewer rotations than vehicle treated mice. **c** PBT434 (30 mg/kg/day for twenty days) administered 24 h following intoxication with MPTP resulted in significantly reduced SNpc neuronal loss (*** *P* < 0.001, One-way ANOVA, Tukey Post Hoc). PBT434-met 30 mg/kg/day (PBT434 without the metal binding site) does not protect against MPTP. **d** PBT434 treatment resulted in improvement in motor performance in the Pole test (* *P* < 0.05, One-way ANOVA, Tukey Post Hoc). UL = unlesioned, VEH = standard suspension vehicle without compound, PBT434-met = analogue of PBT434 without the metal binding

Pharmacokinetic (PK) data showed that PBT434 was orally bioavailable, readily penetrated the blood brain barrier, and was well-tolerated in mice (Additional file 1: Data S3). The effect of PBT434 was initially tested in the mouse 6-OHDA toxin model, where oral PBT434 (30 mg/kg/day) was administered 3 days after the toxin (Fig. 3a, b and Additional file 1: Fig. S4). PBT434 prevented neuronal loss following 6-OHDA, preserving up to 75% of the SNpc neurons remaining (both Nissl and tyrosine hydroxylase (TH) positive neurons) after the initial phase of cell death (*p* < 0.001). While rotational behavior was improved with both L-DOPA (20 mg/kg/day) and PBT434, L-DOPA did not prevent nigral damage.

Subsequent studies were only performed using the MPTP intoxication model, which permitted higher throughput and larger animal numbers per trial. The toxin-induced time course of SNpc cell death caused by MPTP is characterized by initial phase of rapid cell death, followed by a more gradual cell loss continuing up to 21 days after the initial insult [48–51, 65, 86]. We confirmed this pattern of cell loss in our MPTP paradigm using stereological counting of SNpc neurons on days 1, 3, 10 and 21 following administration of toxin (Additional file 1: Fig. S4). To avoid possible interactions with the toxin, PBT434 was administered to MPTP-treated mice one-day post intoxication. Animals in the treatment arm were gavaged once daily with PBT434 or its non-metal binding analog PBT434-met, each at 30 mg/kg/day (a dose which preliminary studies established was well tolerated, Additional file 1: Data S3). PBT434 was tested in 12 separate experiments. On average across these trials, MPTP induced a 44% ± 4% depletion of SNpc neurons. The PBT434-met analog control had no significant effect (Fig. 3c). Motor activity in MPTP treated mice was assessed at 21 days by pole test. We adopted the pole test for the MPTP studies, as this task is sensitive to MPTP-induced denervation [44, 45, 61, 69]. When compared with vehicle alone PBT434 significantly reduced MPTP-mediated motor deficits in the pole test (*p* < 0.001; Fig. 3d).

### Dose-response

**Fig. 4:**
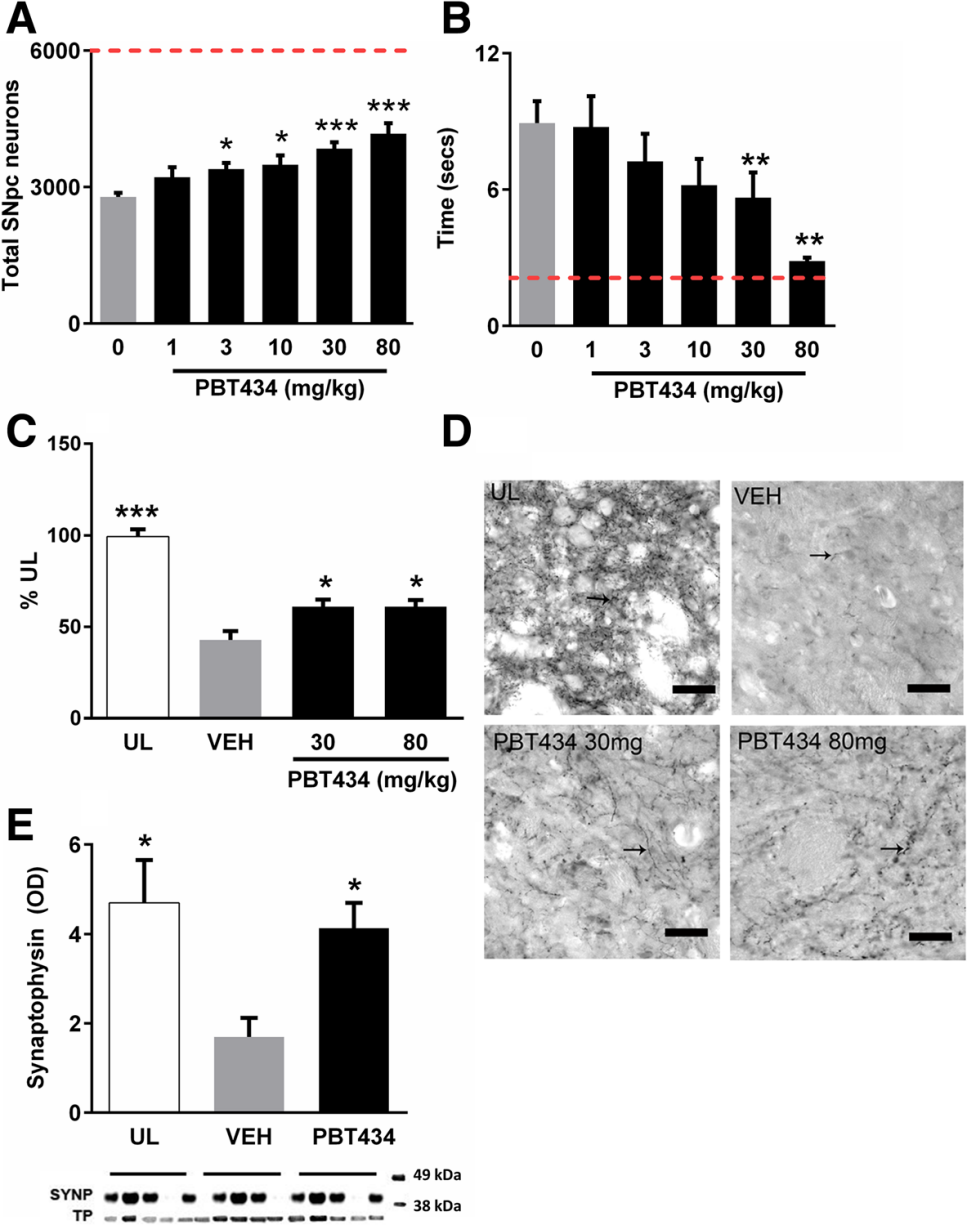
Dose response effects of PBT434 on neuron number and TH - positive varicosities. The effects on SNpc neuron number and motor function in response to escalating dose of PBT434 were assessed. 12–14 week old male C57BL/6 mice were lesioned using MPTP (60 mg/kg) resulting in an average SNpc lesion size of 55%. Treatment with PBT434 at 1,3,10, 30 or 80 mg/kg/day commenced 24 h after induction of lesion. **a** Mean number of SNpc neurons compared with the vehicle treated group (VEH)(±SEM). The proportion of SNpc cells rescued increased with increasing dose of PBT434. 3 mg (*P* < 0.05), 10 mg (*P* < 0.01), 30 mg (*P* < 0.001) and 80 mg/kg/day (*P* < 0.001) doses prevented a significant proportion of cell loss by day 21 (73 animals were studied from two separate MPTP experiments; One-way ANOVA paired with Games-Howell post hoc test). The red dotted line indicates the normal value. **b** Pole test was undertaken at day 20 post intoxication to test motor performance. As the dose of PBT434 increased there was a trend to improved turning behavior compared with the vehicle group while doses 30 mg/kg/day (*P* < 0.05) and 80 mg/kg/day (*P* < 0.01) showed a significant 2.5–3 fold improvement in the time to turn. (One-way ANOVA paired with Games-Howell post hoc test). The dashed line represents the average time taken by unlesioned animals to perform the task. **c** The abundance of tyrosine hydroxylase-positive varicosities in the caudate putamen was assessed by stereology at day 21 following MPTP (*N* = 6–7 animals per treatment). Following MPTP intoxication, TH -positive varicosities were reduced compared with unlesioned mice (* *p* < 0.05. One-way ANOVA, Tukey’s Post hoc comparison). Treatment with 30 or 80 mg/kg of PBT434 significantly increased varicosity abundance compared with untreated mice. **d** Light micrographs of the dorso-lateral tier of the caudate putamen showing individual tyrosine hydroxylase -positive varicosities (arrows), for unlesioned animals (UL), MPTP lesioned vehicle treated (VEH) or following PBT434 treatment (scale bar = 25 μm). **e** Western blots of the dorsal tier of the caudate putamen showed that treatment with PBT434 prevented the decline in levels of the presynaptic marker synaptophysin (SYNP), * *p* < 0.05, One-way ANOVA, Tukey Post hoc comparison). TP = total protein, OD = optical density

In order to establish if a dose-response relationship existed, we administered 1, 3, 10, 30 or 80 mg/kg PBT434 to MPTP-challenged mice for 20 days, as described. SNpc neuron number (Fig. 4a) and motor performance (pole test, Fig. 4b) were assessed for each dose arm at day 21 post-intoxication. The proportion of SNpc cells preserved increased incrementally with increasing dose of PBT434 with significance becoming apparent at 3 mg/kg/day, and maintained at 10, 30 and 80 mg/kg/day. As the dose of PBT434 increased there was a dose-dependent improvement motor performance on the pole test, reaching significance at 30 mg/kg/day, and almost completely rescued at 80 mg/kg/day.

### Nigro-striatal connectivity

Varicosities are the sites where the axons of SNpc neurons synapse with neurons in the caudate/putamen. Typically, in PD and in MPTP lesions there is substantial loss of these synapses. At 21 days after MPTP intoxication, TH- positive varicosities in vehicle treated animals were reduced by more than 50% compared with unlesioned mice (*p* < 0.05). The number of TH- positive varicosities was significantly higher in PBT434-treated animals at both 30 and 80 mg/kg when compared with untreated animals (Fig. 4c, d). In a complementary study, we found that MPTP treatment significantly reduced levels of the presynaptic protein synaptophysin, and this lesion was abolished by treatment with PBT434 (30 mg/kg/day) (Fig. 4e).

### Effects of PBT434 upon toxin-mediated elevation of iron

**Fig. 5:**
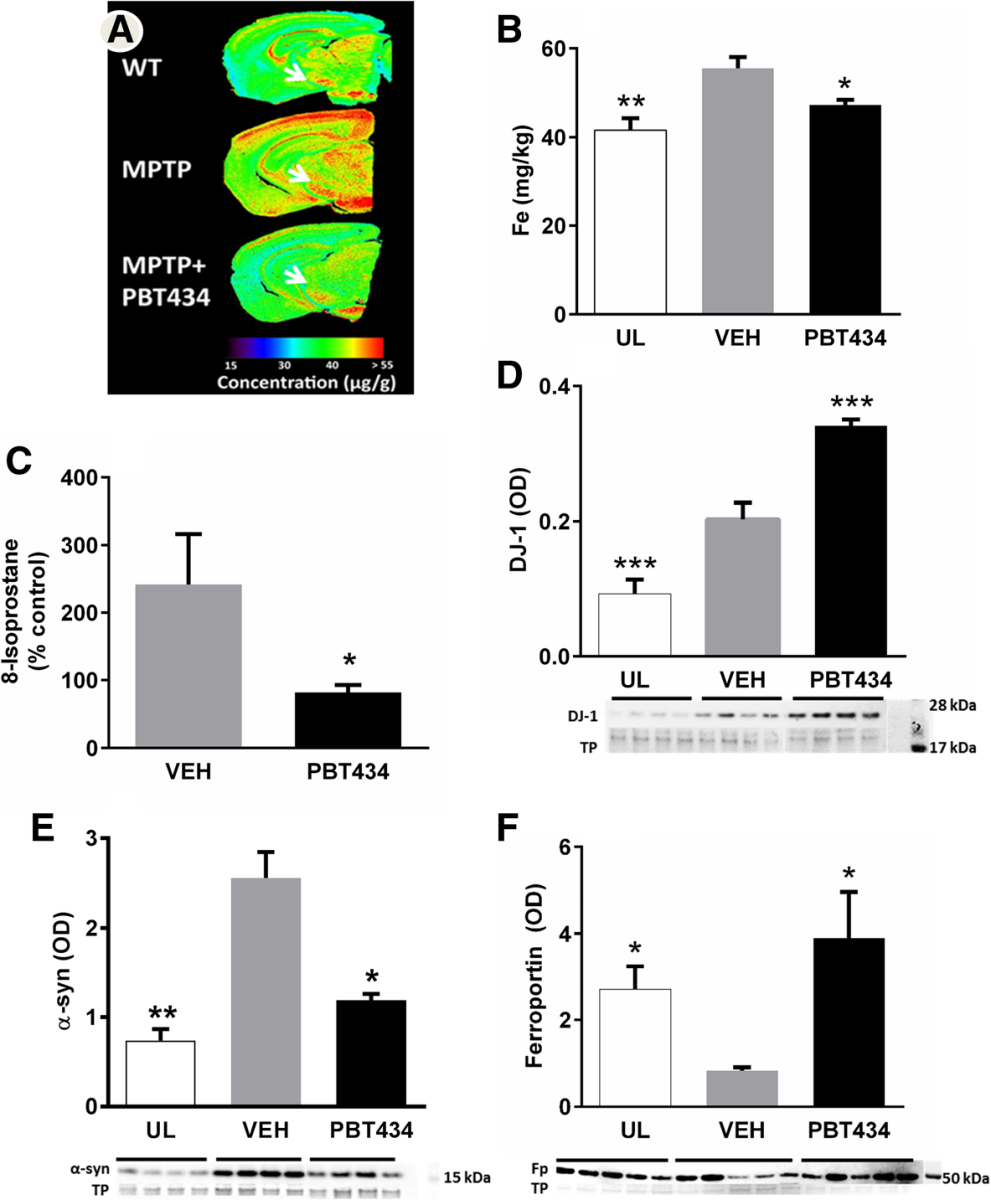
PBT434 improves iron level following MPTP lesion. 12–14 week old male C57BL/6 mice were lesioned using MPTP which resulted in a lesion size 65–70% cell loss by day 21. MPTP lesioned mice were treated with vehicle (VEH) or PBT434 (30 mg/kg/day) from day 1 to day 21. **a** Brain samples collected at day 21 were sectioned and scanned using laser ablation-inductively coupled plasma-mass spectrometry. Representative images show Fe distribution in normal, unlesioned, wildtype (C57BL6) mouse brain, MPTP lesioned brain and MPTP + PBT434 treated brain. The heat map quantifies the level of iron in the SN, which is indicated by the arrow. **b** The concentrations of Fe (mg/kg) in the substantia nigra (SN, includes both compacta and reticulata) of the groups of mice were plotted onto a bar graph. MPTP injury causes a significant elevation in Fe in the SN at day 21 (**p < 0.01, one-way ANOVA, Tukey post hoc) compared with unlesioned mice (UL) which was attenuated by PBT434 (* *p* < 0.05, one-way ANOVA, Tukey post hoc). **c** PBT434 significantly prevented the MPTP induced elevation of 8-isoprostane within SN as measured by ELISA (*P < 0.05, One-way ANOVA, Tukey Post Hoc). **d** Western blot was used to measure levels of levels of DJ-1 in the SN. Levels of DJ-1 were significantly elevated with MPTP treatment in the absence of drug (VEH) and significantly further elevated with PBT434 (TP = Total Protein; OD = optical density; ***P < 0.001, One-way ANOVA, Tukey post hoc). The protein ran at the predicted molecular weight (24 kDa) as can be seen by comparing the position of the proteins with the molecular weight ladder on the right of the image. **e** α-synuclein levels in mice administered MPTP or MPTP + PBT434 (30 mg/kg/day, were compared with unlesioned controls; UL). SN tissue samples were homogenized to form a lysate, which was assayed by Western blot and quantitated by optical density (OD) normalized to total protein (TP, Ponceau). The protein ran at the predicted molecular weight 14 kDa compared to with the molecular weight ladder on the right of the image. In MPTP lesioned mice α-synuclein was significantly elevated by day 21 (**P < 001, one-way ANOVA, Tukey post hoc). α-synuclein protein levels were significantly lower with PBT434 treatment (*P < 0.05, one-way ANOVA, Tukey post hoc) compared with Vehicle treated animals. **f** Western blot of MPTP lesioned mice showed a significant reduction in levels of ferroportin protein which were decreased 21 days after the lesion (*P < 0.05). The protein ran at slightly less the predicted molecular weight 63 kDa compared to with the molecular weight ladder on the right of the image. Ferroportin protein levels were significantly higher with PBT434 treatment (*P < 0.05) compared with the vehicle treated animals but not different to unlesioned mice (one-way ANOVA, Tukey post hoc)

We applied laser ablation inductively-coupled plasma mass spectrometry (LAICPMS) [38, 39, 58] to monitor the level and distribution of iron in the SN of test animals. This technique, while providing more information than manual microdissection, could not discriminate between SN pars reticulata and SNpc. Not to our knowledge reported previously, MPTP challenge caused iron levels to rise in several brain regions 21 days after the MPTP. Iron levels in the SN were elevated by around 25% compared with the control animals at day 21, and were normalized by PBT434 treatment (30 mg/kg/day by oral gavage) (Fig. 5a). Solution phase ICPMS applied to SNpc tissue dissected manually from a separate MPTP/PBT434 cohort confirmed these results, and found that neither MPTP nor PBT434 significantly affected SNpc copper levels (Additional file 1: Figure S5).

### Oxidative stress markers

In MPTP lesioned animals, levels of the oxidative stress marker 8-isoprostane in the SNpc were elevated to over 200% those of the unlesioned controls. 8-isoprostane levels in the corresponding PBT434-treated cohort did not rise significantly above control levels (Fig. 5c). Conversely, DJ-1 levels were significantly increased by MPTP and further elevated in PBT434-treated animals (Fig. 5d).

### Effect of PBT434 upon MPTP-mediated elevation of α -synuclein

MPTP intoxication in wild-type mice has been reported to cause an increase in α-synuclein protein levels in the SNpc [79, 89]. We found that MPTP induced a significant and marked rise in α-synuclein levels in the SNpc that persisted at day 21, and was abolished by concurrent PBT434 treatment (Fig. 5e).

### Effect on ferroportin

In previous work we showed that iron accumulation in an animal model of Parkinsonism was associated with failure of the iron export apparatus [4, 5, 61]. Consistent with previous reports [26], we found that MPTP caused a profound reduction in levels of the iron export protein ferroportin in the SNpc. Treatment with PBT434 (30 mg/kg/day for 20 days), prevented this and ferroportin protein levels remained similar to those of unlesioned animals (Fig. 5f).

### Effect of PBT434 in the α-synuclein transgenic (hA53T) mouse

**Fig. 6:**
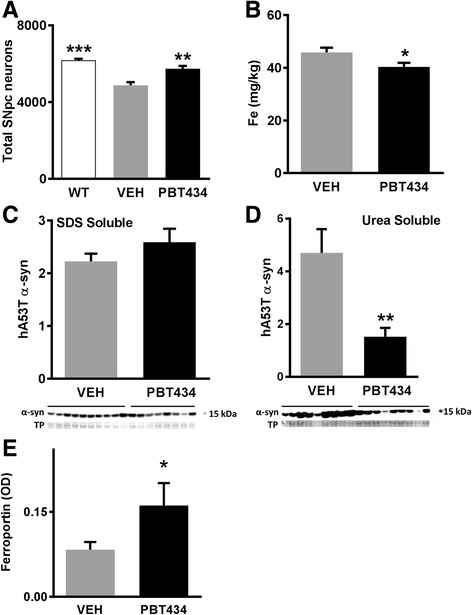
PBT434 modulates α-synuclein transgenic animals. hA53T α-synuclein Tg mice consumed an average of 37 mg/kg/day in animal chow of PBT434 from 4 months of age for 4 months. **a** PBT434 preserved SNpc neurons (** *P* < 0.01, one-way ANOVA, Tukey post hoc); **b** PBT434 decreased SN iron measured by mass spectrometry (*P < 0.05, T-Test). SN tissue samples were homogenized to form a lysate, which was assayed by western blot and quantitated by optical density (OD) normalized to total protein (TP, Ponceau). **c** PBT434 did not reduced levels of the SDS soluble fraction of α- synuclein in the SN (Western blot, urea soluble fraction); **d** PBT434 reduced levels of the urea soluble fraction of α- synuclein in the SN (** *p* < 0.01; T-Test); E) PBT434 treatment increased SN ferroportin levels (*P < 0.05, T-Test)

We tested whether the neuroprotective effects of PT434 could be reproduced in a genetic model of PD. The hA53T mutant α- synuclein transgenic mouse has a subtle disease phenotype [12, 14, 31, 35, 72]. At 8 months of age, the hA53T mice exhibit decreased locomotion in the open field test compared with wild type animals, and hindlimb clasping behavior (Additional file 1: Figure S6), indicative of striatal damage. This is accompanied by a modest but significant decrease in the number of nigral neurons between 4 and 8 months of age. Long term (4 months) treatment with PBT434 incorporated into the animal feed (to achieve an average dose of 30–37 mg/kg/day) from 4 months of age significantly preserved SNpc neuron number (Fig. 6a) accompanied by increased total movements in the open field test and reduced clasping behavior (Additional file 1: Figure S6). In the Tg mice at 8 months, four months of PBT434 treatment reduced SN iron levels by 15% (Fig. 6b) PBT434 treatment did not alter the levels of soluble α- synuclein (Fig. 6c). but significantly decreased the nigral insoluble (urea extracted) α- synuclein (Fig. 6d). and significantly increased nigral ferroportin levels (Fig. 6e).

### Cerebral spinal fluid (CSF) biomarkers

**Fig. 7:**
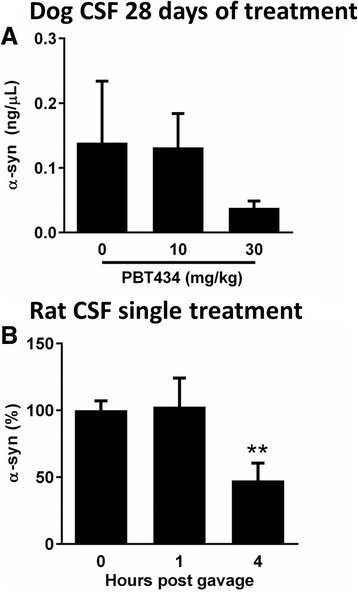
α-synuclein levels in CSF of dogs and rats following PBT434 treatment. α-synuclein was collected and quantified from CSF of dogs and rats following PBT434 treatment. **a** α-synuclein levels of CSF collected from dogs following 28 days exposure to PBT434 at various doses. α-synuclein levels detected by enhanced Western blot decline in the 10 mg/kg, did not reach significance (one-way ANOVA, Tukey post hoc). **b** Cannula were implanted into the lateral ventricles of wild type rats and CSF was sampled before and after gavage with 30 mg/kg/day PBT434. Western blot for the presence of showed a significant decrease in α-synuclein at 4 h but not at 1 h (* *P* < 0.05, one-way ANOVA, Tukey post hoc)

In a small exploratory study, CSF collected *post-mortem* from dogs undergoing a 28 day dose-tolerability study of PBT434 was assayed for soluble α-synuclein a dose dependent trend (non-significant) in the reduction of α-synuclein was observed (Fig. 7a). To further investigate this phenomenon in a more controlled environment, cannula were surgically implanted into the lateral ventricles of rats (*n* = 10). CSF was sampled before and after gavage with 30 mg/kg PBT434 and analyzed by Western blot for the presence of α-synuclein. At four hours but not at one hour, α-synuclein levels were significantly lower than baseline (*p* = 0.05, Fig. 7b).

## Discussion

PBT434 is a novel, orally bioavailable, moderate iron affinity 8-hydroxyquinazolinone which is being developed for treatment of Parkinsonian conditions. We adapted commonly used Parkinsonian toxin models and the hA53T α-synuclein transgenic mouse to investigate the therapeutic potential of PBT434 to slow or prevent progressive neurodegeneration in PD. We found that PBT434 preserved SNpc neuron number in animal models of SNpc degeneration and synucleinopathy which translated to improved motor function accompanied by reduced levels of α-synuclein and a reduction in markers of oxidative stress. The observation that the analog of PBT434, (PBT434-met), in which the metal binding site was blocked, had no protective effect, is consistent with the proposed metal-centric mechanism of action.

Although there is an accumulation of iron in the neurons of the SN in PD [71], it may be simplistic to characterize the condition as a disease of iron overload analogous to hemochromatosis or thalassemia in which potent chelators are required for clinical effect [75]. Evidence suggests rather, that subtle alterations to mechanisms maintaining metal homeostasis are disrupted, leading to anatomically localized deficits and surpluses [4–6, 61]. Inevitably this results in an increase in the weakly bound or labile metal pool [7, 8], including iron, a potent generator of free-radicals. Recent findings have placed iron close to the center of pathological events [5, 21]. For instance, it has been recently demonstrated that cellular reactive oxygen species production by α-synuclein oligomers is entirely dependent on the presence of free metal ions, as addition of metal chelators could block oligomer-induced reactive oxygen species production and prevent oligomer-induced neuronal death [23]. Furthermore, oxidative stress, a feature of both disease and aging is now understood to be sufficient in of itself to engender the deterioration in the performance of the iron trafficking apparatus which is manifested in aberrant iron distribution [21, 68, 76].

With a Kd around 10^10^ the affinity of PBT434 for iron is considered moderate compared with physiological iron ligands such as transferrin (Kd 10^23^) [3] and orders of magnitude lower than iron chelators in clinical use such as deferiprone Kd 10^35^) [55]. The significantly lesser efflux of iron from cultured cells engendered by PBT434 compared with deferiprone reflects the widely differing Kds and physicochemical properties of the two drugs. As a corollary, no significant effect on global brain iron levels in normal intact mice was observed within the effective dose range of PBT434.

We assessed the efficacy of PBT434, administered as a single daily oral dose, in attenuating the neurotoxic cascade following intoxication with the Parkinsonian toxins 6-OHDA and MPTP. As SNpc neurons continue to die for weeks after intoxication with both 6-OHDA and MPTP efficacy [48, 50, 51, 65, 86], we elected to evaluate the extent of preservation of SNpc neurons at 21 days as the primary measure of efficacy. MPTP has the advantage of being able to produce larger numbers of uniformly lesioned animals, therefore most the studies were undertaken using the MPTP model. By delaying administration of PBT434 until the toxin is known to have been cleared [47, 90] we obviated the possibility of false positive results caused by incidentally neutralizing the toxin, by blocking its uptake, or by preventing its conversion to MPP+ [56]. The 6-OHDA toxin has similar potential for interactions with test compounds, therefore PBT434 administration was delayed until 72 h after intra-nigral injection of 6-OHDA. 6-OHDA lesion size being directly related to the rotational behavior (Additional file 1: Figure S4). Further stringency was introduced by selecting only those mice that rotated 200–450 times an hour. The presence of an MPTP-induced lesion is related to motor dysfunction although unlike 6-OHDA, no direct correlation has been shown between lesion size and motor performance [45, 69]. We found the MPTP-affected brain to be sensitive to dose of PBT434; incremental improvements in SNpc neuron viability reflected in similarly dose-dependent improvements in motor function (Fig. 4). While increasing survival of SNpc neurons is important, in order for function to be maintained, connectivity between these neurons and their synaptic partners in the striatum is required. PBT434 preserved nigro-striatal synaptic circuitry, reflected in the abundance of terminal varicosities. The maintenance of nigro-striatal synaptophysin protein at levels seen in intact animals supports the conclusion that preservation of connectivity is likely a major contributor to the improvement in motor function observed with PBT434.

As ferroportin is the primary iron exporting protein, the precipitous drop in ferroportin levels following the MPTP insult may be sufficient to explain the rise in SNpc iron [60]. Presumably, the effect of PBT434 in sustaining ferroportin levels is a beneficial consequence of its neuroprotective effect although a direct effect cannot yet be ruled out.

It has been reported that iron, copper and dopamine under oxidizing conditions each foster the aggregation of α-synuclein and the formation of toxic soluble oligomeric species [19, 43, 63]. The sharp rise in α-synuclein levels following MPTP treatment is well documented [89]. We speculate that in the MPTP model PBT434 modulates α-synuclein expression via the iron responsive element on the 5′ untranslated region of its mRNA by sequestering weakly bound cellular iron. It has been reported that α-synuclein itself has ferri-reductase activity capable of reducing Fe III to Fe II which lends further weight to the argument that its expression is likely finely tuned to the presence of iron [22]. The reduction in α- synuclein in the A53T mouse cannot be explained by altered expression, as the A53T transgene does not encode the IRE sequence which would permit it to respond to alterations in cellular iron levels. As noted previously, over the course of treatment the level of nigral iron in the transgenic mice did decline by a modest but significant 15%. We hypothesize that this reflects an enlarged pool of labile or weakly bound iron previously identified as a feature of the Parkinsonian brain [54], which may interact aberrantly with α-synuclein to promote aggregation and deposition.

We postulated that if the ability of PBT434 to prevent the elevation in α-synuclein levels following MPTP in wild type mice was due to its effect on the labile iron pool, that same effect might also be detectable in healthy animals. Examination of samples of post-mortem CSF from healthy dogs treated with PBT434 for 28 days showed a significant dose-dependent reduction in α-synuclein, an effect also observed in rats fitted with an indwelling ventricular catheter, consistent with the hypothesis that PBT434 modulates α-synuclein expression at the translational level. This finding raises the possibility that CSF α-synuclein may be of value as a marker of target engagement in the clinical setting. There is considerable debate and some justifiable skepticism surrounding the use of biomarkers like CSF α-synuclein as indices of disease progression [52], however, such biomarkers, especially if they are demonstrably linked to drug mechanism of action, may be judiciously applied to the evaluation of target engagement for new drugs, assisting in the early phases of clinical development.

The potential for oxidative damage is increased when the normally tight control over the trafficking of redox active metals is impaired. Such impairment may be related to altered activity of the metal trafficking apparatus [8, 80] or more indirectly through impaired function of iron dependent enzymes of the (mitochondrial) oxidative phosphorylation apparatus and enzymes such as hypoxia inducible factor (HIF) which have roles in the adaptation to hypoxic or oxidative stress [84]. The marked reduction in 8-isoprostane levels observed in the MPTP treated mice following treatment with PBT434 indicates that its anti-oxidant properties translate to the in vivo milieu.

Defects in the gene encoding the protein deglycase DJ-1 are a cause of autosomal recessive early-onset Parkinson’s disease [37]. In pro-oxidative conditions, DJ-1, also known as Park7, has been shown to inhibit the aggregation of α-synuclein, to function as a redox-sensitive chaperone [83], as a sensor for oxidative stress and as a mitochondrial pacemaker minimizing oxidative stress associated with dopamine secretion [37]. It has also been shown to bind metals and protect against metal mediated toxicity [13]. The effect of PBT434 upon this multifaceted protein is particularly intriguing because intoxication with MPTP causes a substantial rise in DJ-1 which is further enhanced by treatment with PBT434. Further investigation will reveal the role of DJ-1 in the response to MPTP and where that pathway intersects with the mechanism of action of PBT434. In vitro, DJ-1 has been shown to inhibit the toxicity of A53T α-synuclein [91, 92]. In our hands, by the age of 8 months, there was as yet scant evidence of acute oxidative damage in the A53T mice similar to the MPTP model, nor did we observe alterations to the levels of DJ-1. Literature sources suggest that any such damage may be more subtle, manifested in oxidation of mitochondrial proteins [66] and in particular in post translational modifications of α-synuclein itself such as oxidation or nitration [88].

## Conclusions

Genetic and experimental evidence strongly implicate α-synuclein in the etiology of Parkinson’s disease, recommending this protein as a plausible target for potential disease modifying therapies. As understanding of the role of iron in the pathological process in PD evolves, evidence is emerging that α-synuclein levels may be modulated by selective targeting of this ubiquitous biometal. PBT434 was developed to exploit this therapeutic niche and in addition to its potential utility in the clinic will be a valuable tool for studying the role of metals in modulating α-synuclein levels, the role of oxidative stress as an initiator and perpetuator of the nigral lesion and the involvement of other components of the neuronal iron trafficking apparatus. Treatments currently available for PD and the atypical Parkinsonian conditions at best provide limited symptomatic relief and do not alter disease progression. The beneficial effects of PBT434 on motor function, neuropathology and biochemical markers of disease state in three different animal models of PD suggest disease modifying potential.

## Additional file

Additional file 1: Data S1. Potentiometric determination of PBT434 affinity for Fe(III), Fe(II) Cu(II) and Zn(II) Ions. **Figure S2.** Effect of PBT434 on brain iron and peripheral markers of iron metabolism. **Data S3.** Pharmacokinetics, pharmacodynamics and safety. **Figure S4.** Experimental schema. **Figure S5.** Fe and Cu levels in SNpc (solution ICP-MS). **Figure S6.** Effect of PBT434 on the phenotype of the transgenic hA53T. (DOCX 4044 kb)

## Abbreviations

6-OHDA: 6-hydroxydopamine
CPM: Counts per minute
CPu: Caudate putamen
CSF: Cerebral spinal fluid
DA: dopamine
Kd: dissociation constant
L-DOPA: L-3,4-dihydroxyphenylalanine
MPTP: 1-methyl-4-phenyl-1,2,3,6-tetrahydropyridine
PBT434: 8-hydroxyquinazolin-4(3H)-one
PBT434-met: PBT434 in which the metal binding site was blocked
PD: Parkinson’s disease
PK: Pharmacokinetic
RFU: Relative fluorescent units
SNpc: substantia nigra pars compacta
TH: tyrosine hydroxylase
TP: total protein
