# Correction to: The novel compound PBT434 prevents iron mediated neurodegeneration and alpha-synuclein toxicity in multiple models of Parkinson’s disease

**DOI:** 10.1186/s40478-021-01258-8

**Published:** 2021-09-29

**Authors:** David I. Finkelstein, Jessica L. Billings, Paul A. Adlard, Scott Ayton, Amelia Sedjahtera, Colin L. Masters, Simon Wilkins, David M. Shackleford, Susan A. Charman, Wojciech Bal, Izabela A. Zawisza, Ewa Kurowska, Andrew L. Gundlach, Sheri Ma, Ashley I. Bush, Dominic J. Hare, Philip A. Doble, Simon Crawford, Elisabeth C. L. Gautier, Jack Parsons, Penny Huggins, Kevin J. Barnham, Robert A. Cherny

**Affiliations:** 1 The Florey Institute of Neuroscience and Mental Health, The University of Melbourne, Melbourne, VIC 3010 Australia; 2 Prana Biotechnology Ltd, Parkville, VIC 3052 Australia; 3 Centre for Drug Candidate Optimisation, Monash Institute of Pharmaceutical Sciences, Monash University, Parkville, VIC 3052 Australia; 4 The Institute of Biochemistry and Biophysics, Polish Academy of Sciences, Warsaw, Poland; 5 Elemental Bio-Imaging Facility, The University of Technology Sydney, Broadway, Ultimo, NSW 2007 Australia; 6 Australia Electron Microscope Unit, School of Biosciences, The University of Melbourne, Melbourne, VIC 3010 Australia; 7 Bio21 Institute and Department of Pharmacology and Therapeutics, The University of Melbourne, Melbourne, VIC 3010 Australia

## Correction to: Acta Neuropathologica Communications (2017) 5:53 https://doi.org/10.1186/s40478-017-0456-2

Following publication of the original article [1], the author identified an error in Fig. 4E. The data and statistics were correct, but the synaptophysin blot was incorrect.

The incorrect (Fig. 1) and correct figure (Fig. 2) are shown in this correction article.

**Fig. 1:**
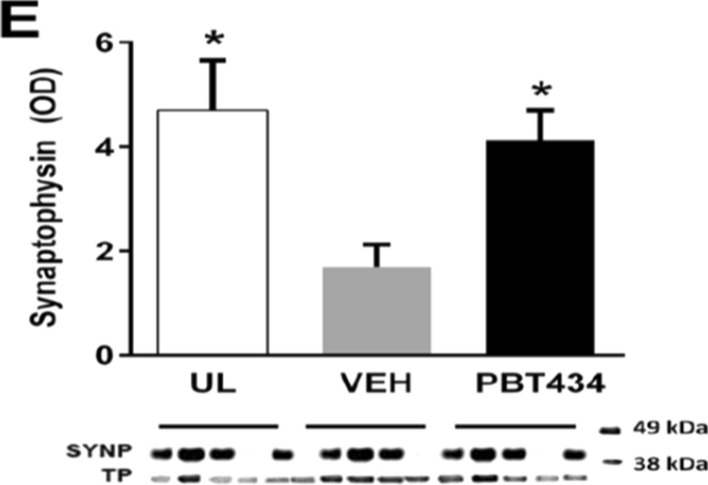
Incorrect version of Fig. 4E as originally published

**Fig. 2:**
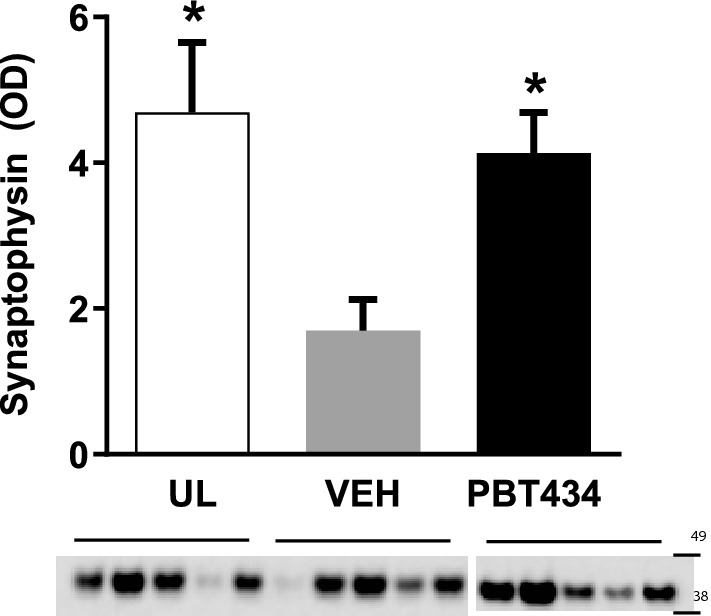
Correct version of Fig. 4E
