# Supplementary material for: The novel compound PBT434 prevents iron mediated neurodegeneration and alpha-synuclein toxicity in multiple models of Parkinson’s disease
Source: Acta Neuropathol Commun. 2017 Jun 28;5:53. doi: 10.1186/s40478-017-0456-2 (PMC5490188; doi:10.1186/s40478-017-0456-2)
Supplement: Additional file 1: Data S1. — Potentiometric determination of PBT434 affinity for Fe(III), Fe(II) Cu(II) and Zn(II) Ions. Figure S2. Effect of PBT434 on brain iron and peripheral markers of iron metabolism. Data S3. Pharmacokinetics, pharmacodynamics and safety. Figure S4. Experimental schema. Figure S5. Fe and Cu levels in SNpc (solution ICP-MS). Figure S6. Effect of PBT434 on the phenotype of the transgenic hA53T. (DOCX 4044 kb) [file 40478_2017_456_MOESM1_ESM.docx]

**Supporting information**

**“The novel compound PBT434 prevents iron mediated neurodegeneration and alpha-synuclein toxicity in multiple models of Parkinson’s disease.”**

**Supplemental Data S1: Potentiometric determination of PBT434 affinity for Fe(III), Fe(II) Cu(II) and Zn(II) ions**

**Acid-base properties of PBT434**

The p*K*_a_ values obtained by potentiometric and UV-vis titrations are presented in Table 1. Potentiometry is the method of choice due to its accuracy, but is indirect and good practice requires that such data are corroborated by a direct method, such as UV-vis (1)The UV-vis titrations provided constants which are only slightly different from the potentiometric ones, due to differences in electrode calibration (2) PBT434 released two hydrogen ions in the course of acid-base titrations, one in the weakly basic region, p*K*_a_ ~6.1 and , p*K*_a_ ~9.7. The difference of more than 3 log units between these values, confirmed by both methods, indicates that the individual deprotonations are localized to two separate sites of the molecule.

**Table 1.** Protonation constants (p*K*_a_ values) of PBT434 obtained by potentiometry and UV-vis spectroscopy. Statistical errors on the last digits of constant values are given in parentheses.

| **Reaction** | **Potentiometry (p*K*_a_)** | **UV-vis** |
| --- | --- | --- |
| H_2_L = HL + H^+^ | 6.12(1) | 6.277(3) |
| HL = L + H^+^ | 9.685(4) | 9.45(3) |
| H_2_L = L + 2H^+^ | 15.804(7) | 15.73(3) |

**Metal ion binding to PBT434**

For all four metal ions two stoichiometries were found, ML and ML_2_. The differences in affinity of binding between the first and the second PBT434 molecule to the metal ion were very high for Fe(III) and Cu(II), smaller for Fe(II) and very small for Zn(II). In terms of overall affinities, the Cu(II) binding was the strongest, followed by Fe(III) for the first PBT434 molecule and by Zn(II) for the second molecule. The Fe(II) complexes were the weakest of all studied metal ions in both cases. As indicated by data in Table 2, the realistic stoichiometries for physiological conditions are 1:1 for Fe(III) and Cu(II), but Fe(II) and also Zn(II) complexes are weaker and probably irrelevant biologically.

The absolute stability constants provided in Table 2 have to be corrected according to the protonation pattern of PBT434. At pH 7.4 its main form is monoprotonated and the metal ion has to compete one hydrogen ion out of the PBT434 molecule in order to form a complex. Therefore, the absolute constants for complexes must be corrected for this protonation, by ca. 2.3 log units per bound PBT434. As a result, the approximate affinity of BPT-434 for Fe(III) and Cu(II) is of the order of 10^-10^ M, while for Zn(II) it is 10^-6^ - 10^-7^ M and for Fe(II) it is ca. of 10^-5^ M.

**Table 2.** Protonation constants and stability constants (log β values and conditional dissociation constants at pH 7.4) of metal ion complexes of PBT434 at I = 0.1 M (KNO_3_) and 25 °C. Standard deviations on the last digits are given in parentheses.

| **Reaction** | **Stability constant (log *K*)** | | | |
| --- | --- | --- | --- | --- |
|  | **Fe(III)** | **Fe(II)** | **Cu(II)** | **Zn(II)** |
| M + L = ML  (log *K*_1_) | 11.86(1) | 7.06(1) | 12.42(2) | 8.57(2) |
| M + 2L = ML_2_  (log(*K*_1_×*K*_2_)) | 15.30(3) | 11.06(4) | 19.33(4) | 16.54(2) |
| ML + L = ML_2_  (log*K*_2_) | 3.44 | 4.0 | 6.91 | 7.97 |
| *K*_1_/*K*_2_ | 2.6×10^8^ | 1.1×10^3^ | 3.2×10^5^ | 4 |
|  | **Apparent association / dissociation constant at pH 7.4** | | | |
| M + L = ML | 10^9.6^ M^-1^/0.3 nM | 10^4.7^ M^-1^/18 µM | 10^10.1^ M^-1^/80 pM | 10^6.3^ M^-1^/0.55 µM |

**Supplemental Figure S2: Effect of PBT434 on brain iron and peripheral markers of iron metabolism.**

12-14 week C57BL/6 male mice were treated for 21 days by oral gavage with vehicle PBT434 at 30 mg/kg/day (N=4) and were compared to untouched controls (CONT) and vehicle treated controls (VEH). The mice were deeply anaesthetised and blood obtained by cardiac puncture which was then analyzed for markers of iron metabolism; A) Hematocrit, B) Hemoglobin, C) Serum B12 D) Serum iron, E) Liver Iron. The samples were analyzed by a veterinary pathology service (Gribbles Veterinary Australia, accreditation to ISO/IEC 17025 conferred by the National Association of Testing Authorities and certification to AS/NZS 9001: 2008 conferred by Lloyd's Register Quality Assurance).

In separate groups mice, they were treated with VEH (Vehicle only no PBT434), 30, 45 and 60 mg/kg/day of PBT434(N=4). The mice were deeply anaesthetised, the blood removed from the vessels of the brain by perfusion with 30 ml of PBS via the heart. Brains were digested in acid diluted and then total brain iron was measured by liquid phase inductively mass spectrometry ( ICPMS, Varian UltraMass 700 Varian, Australia). No significant differences were observed at any dose (one-way ANOVA Tukey post hoc).

**Supplemental Figure S2.**


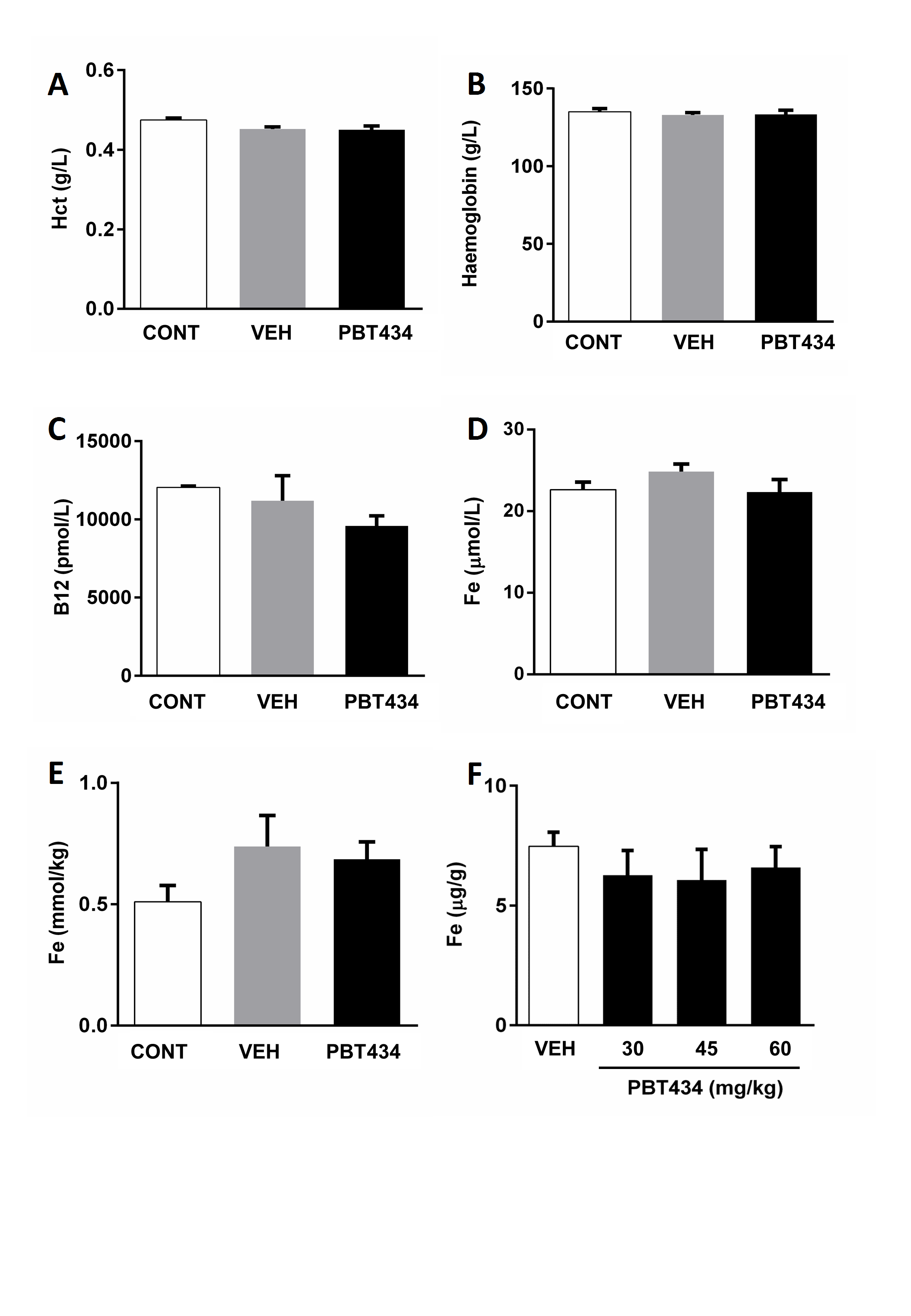


**Supplemental Data S3. Pharmacokinetics, pharmacodynamics and safety**

**Rat pharmacokinetic studies:** Rat pharmacokinetic studies were conducted in accordance with the Australian Code of Practice for the Care and Use of Animals for Scientific Purposes and were approved by the Monash Institute of Pharmaceutical Sciences Animal Ethics Committee. PBT434 was administered orally by gavage to fasted, male Sprague Dawley rats (6 – 8 weeks old) and blood samples were collected and processed as previously described (3). The formulation (1 mL) was an aqueous suspension prepared in a standard suspension vehicle containing 0.9% sodium chloride, 0.5% sodium carboxymethylcellulose, 0.5% benzyl alcohol and 0.4% Tween 80.

**Mouse brain uptake studies:** PBT434 was administered orally by gavage (100 µL per 25 g mouse) to 2 month old C57BL/6 male mice, daily for ten consecutive days. The formulation was prepared in in the same vehicle as described for the rat studies. At 30 and 240 minutes post-dose on day ten (n = 2 mice per time point) mice were anaesthetized with sodium pentobarbitone (100 mg/kg sodium pentobarbitone) diluted in 0.9% saline and 1 U/mL heparin. Blood was withdrawn by cardiac puncture into a heparanized syringe and transferred to Eppendorf tubes containing heparin. Blood samples were centrifuged and aliquots of plasma collected for analysis. Brains were removed and placed into pre-weighed tubes and stored along with the plasma samples at -80°C.

**Bioanalysis of plasma and brain samples:** Brains were homogenized in three parts (by weight) water containing 4 g/L potassium fluoride and 0.1 M EDTA, on ice. PBT434 concentrations were quantified in plasma and brain samples by LC-MS (Micromass Quattro Premier) against calibration standards prepared in blank plasma or brain homogenate. Samples and standards were prepared by precipitation with acetonitrile, followed by centrifugation and analysis of the supernatant. The lower limit of quantitation was 0.5-5 ng/mL in plasma and 50 ng/g in brain homogenate. Stability in brain homogenate was over the processing period was confirmed.

**Results:** PBT 434 is soluble (>1 mg/mL) under pH conditions in the GI tract (i.e. pH 2 and 6.5) and is rapidly absorbed following oral administration. Maximum plasma concentrations (approx. 5955 ng/mL) occur 15 - 30 minutes after a single dose of 30 mg/kg to male Sprague Dawley rats (average data for n=2 animals). Plasma concentrations remained above 2 ng/mL for at least 24 hours post-dose suggesting good exposure after oral administration to rats (average AUC_0-inf_ = 10, 001 µg.h/L). The apparent elimination half-life following oral administration was approximately 3 hours. Similarly, good exposure was observed in C57BL/6 mice following repeated oral dosing, once daily for 10 days at 30 to 120 mg/kg/day. Concentrations of PBT434 in plasma and brain tissue, determined at 30 and 240 minutes after the last dose, increased with increasing dose level with brain-to-plasma ratios remaining relatively consistent (2.1 to 3.9) across the dose range.

**Safety:** Mice treated with up to 60mg/kg PBT434 for 3 weeks remained healthy in appearance and behaviourally normal. PBT434 (30mg/kg, 21days) had no effect upon electrolytes, urea, creatinine, albumin, bilirubin, B12, liver metals, serum metals (Fe, Cu, Zn, Mg), red cell count, hematocrit, serum ceruloplasmin, serum transferrin or brain ceruloplasmin. No significant hits were obtained in an extensive enzyme and receptor profiling screen. Results from four *in vitro* genotoxicity studies, Ames test, chromosome aberration in CHO cells and chromosome aberration and micronucleus in human peripheral lymphocytes, conducted in compliance with ICH and OECD guidelines, demonstrate the absence of significant genotoxic risk with PBT434. The IC50 of PBT434 in the *in vitro* hERG ion channel test suggests a wide safety margin for pro-arrhythmic potential.

**Supplemental Figure S4: Experimental schema.**

**Supplemental Figure S4 : A)** Schematic diagram illustrating the treatment schedules for drug evaluation in the MPTP and 6-OHDA models. PBT434 was tested in both the 6-OHDA and MPTP models in 12-14 week old male C57BL/6 mice. The compound was delivered after the toxin had been metabolized and cell death had commenced. In the MPTP protocol, mice were administered 4 injections of MPTP 2 hours apart on the same day; oral gavage of PBT434 commenced approximately 24 hours after the last MPTP injection. Behavioural assessment was performed at 20-21 days after which the mice were perfused with PBS and the brains removed for analysis. In the 6-OHDA protocol toxin was administered unilaterally by stereotactic intra-nigral injection; at day 3 animals were screened for a sufficient lesion size and then randomly assigned to a drug treatment group or vehicle treated sham. Behavioural assessment was performed at 21 days, after which the mice were perfused and the brains removed for analysis.


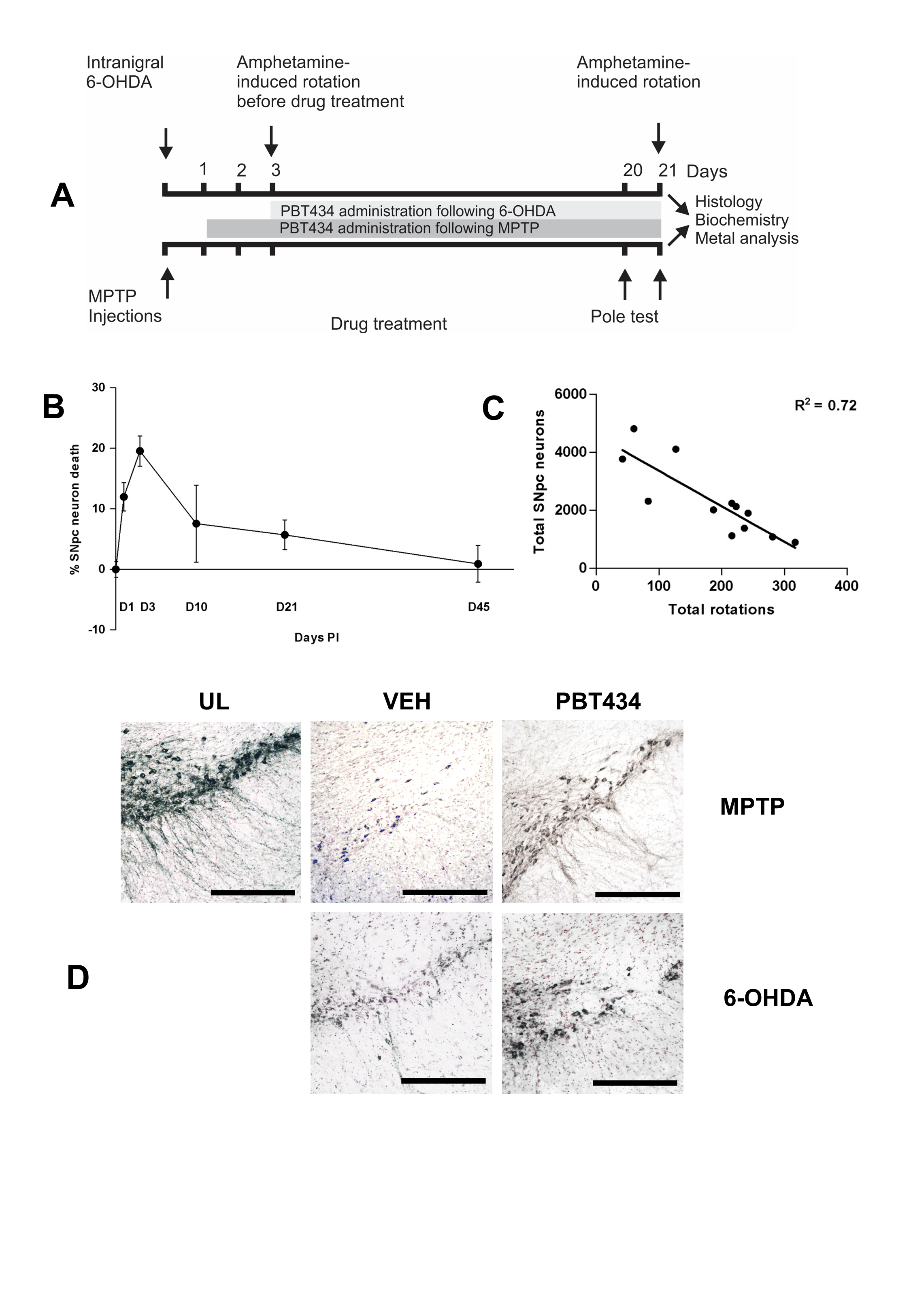


**B)** Time-course of SNpc neuronal cell death following MPTP. Animals were administered MPTP and killed at various time points (N=5-6 mice per time point). The total number of neurons surviving was assessed using stereology. The % cells killed at any time point was calculated by subtracting the number of neurons present at each time point from the number present at the preceding time point (i.e. % killed at Day 3 = N cells present at day 1 - N at Day 3

**C)** Calibration curve correlating SNpc neuron number with rotational behaviour in the 6-OHDA model. The graph was generated by plotting the total number of rotations produced at day 3 against the number of cells surviving at day 21 in individual animals.

**D)** Photomicrographs show PBT434 protects against MPTP-induced and 6-OHDA SNpc neuron loss (tyrosine hydroxylase +ve ). The images show the unlesioned mouse (UL), lesioned vehicle control (VEH) and the lesioned and PBT434 treated mice (scale bar = 500µm).

**Supplemental Figure S5: Fe and Cu levels in SNpc (solution ICP-MS)**





**Supplemental Figure S5.** 12-14 week C57BL/6 male mice were injected with MPTP and were dosed with vehicle (VEH) or PBT434 (30m/kg) for 20 consecutive days substantia nigra (SN) samples were manually dissected (N=6-7 per group), weighed then were prepared for solution ICP-MS to detect iron (Fe) and copper (Cu) levels. MPTP induced a significant increase in nigral Fe (*** P<0.0001, one-way ANOVA Tukey post hoc) compared to unlesioned control animals (UL) seen at 21 days post MPTP treatment. Treatment with PBT434 (30m/kg) prevented the Fe elevation (***P<0.0001, one-way ANOVA Tukey post hoc). No significant difference in nigral Cu levels were detected after MPTP (one-way ANOVA).

**Supplemental Figure S6:**

**Supplemental Figure S6:** hA53T mice were treated from 4 months of age for 4 months with PBT434 that had been incorporated into the animal feed (to achieve an average dose of 30-37mg/kg/day). At 8 months of age, PBT434; A) Reduced incidence of clasping (Chi squared, P<0.001). B) preserved total movements in the open field test (**P< 0.01, *P<0.05, one-way ANOVA, Tukey post hoc).

**References**

1. Szakacs Z, Kraszni M, Noszal B. Determination of microscopic acid-base parameters from NMR-pH titrations. Anal Bioanal Chem. 2004;378(6):1428-48.

2. Zawisza I, Rozga M, Bal W. Affinity of copper and zinc ions to proteins and peptides related to neurodegenerative conditions (A beta, APP, alpha-synuclein, PrP). Coord Chem Rev. 2012;256(19-20):2297-307.

3. Coteron JM, Marco M, Esquivias J, Deng X, White KL, White J, et al. Structure-guided lead optimization of triazolopyrimidine-ring substituents identifies potent Plasmodium falciparum dihydroorotate dehydrogenase inhibitors with clinical candidate potential. J Med Chem. 2011;54(15):5540-61.
